# Ketone Ester Attenuates Thoracic Aortic Aneurysm and Dissection by Suppressing Ferroptosis

**DOI:** 10.3390/cells15090829

**Published:** 2026-05-01

**Authors:** Sanjiv Shrestha, Yang Wu, Jian Li, Xin Du, Ping Song

**Affiliations:** Institute for Biomedical Sciences, Georgia State University, 100 Piedmont Ave SE, Atlanta, GA 30303, USA; sshrestha7@student.gsu.edu (S.S.); dawn0722@gmail.com (Y.W.); jli73@gsu.edu (J.L.); xdu7@student.gsu.edu (X.D.)

**Keywords:** thoracic aortic aneurysm and dissection, ferroptosis, ketone ester, β-hydroxybutyrate, glutathione peroxidase 4, heme oxygenase 1

## Abstract

**Highlights:**

**What are the main findings?**

- Supplementing with ketone esters significantly reduces the incidence of BAPN-induced TAAD and mortality in mice.
- β-OHB suppresses vascular smooth muscle cell ferroptosis through GPX4 induction and HO-1 reduction.

**What are the implications of the main findings?**

- Ketone therapy may serve as an effective preventive strategy for managing aortic pathologies.
- Targeting ferroptotic cell death of vascular smooth muscle cells in the aortic media might provide a novel therapeutic approach.

**Abstract:**

Thoracic aortic aneurysm and dissection (TAAD) is a life-threatening vascular disease lacking therapies that target underlying cell death pathways. Ferroptosis, an iron-dependent form of lipid peroxidation-driven cell death, has emerged as a key mechanism in vascular remodeling. We investigated whether exogenous ketosis induced by ketone ester (KE) supplementation can suppress ferroptosis and prevent TAAD. TAAD was induced in C57BL/6 mice using β-aminopropionitrile (BAPN). A subset of these mice received KE [(R)-3-hydroxybutyl (R)-3-hydroxybutyrate, 20 g/L] in their drinking water starting on day 15 of the BAPN treatment. Human aortic smooth muscle cells (HASMCs) were treated with the GPX4 inhibitor Ras-Selective Lethal 3 (RSL3) and β-hydroxybutyrate (β-OHB) to investigate ferroptotic markers, lipid peroxidation, and labile iron levels. KE supplementation significantly reduced TAAD incidence (69% → 43%) and improved survival rate (52% → 73%), while preserving aortic structure and reducing elastic fiber fragmentation. Transcriptomic analyses of human TAAD datasets (GSE153434 and GSE52093) and single-cell RNA sequencing data (GSE155468) revealed ferroptosis signatures characterized by decreased *GPX4* and increased expression of iron metabolism genes. Mechanistically, KE suppressed BAPN-induced iron accumulation and lipid peroxidation in vivo. In HASMCs, β-OHB inhibited ferroptosis induced by GPX4 inhibition, decreasing lipid peroxidation and labile iron levels. KE restored GPX4 and SLC7A11 expression while suppressing HO-1 in vivo, with effects dependent on Nrf2 signaling in vitro. In summary, ketone ester supplementation protects against TAAD by inhibiting VSMC ferroptosis via GPX4 induction and HO-1 suppression, highlighting a potential therapeutic strategy for aortic disease.

## 1. Introduction

Thoracic aortic aneurysms and dissections (TAAD) are life-threatening conditions characterized by the progressive dilation and weakening of the thoracic aortic wall, which can lead to acute dissection (tearing of the inner aortic wall) or rupture [1,2,3,4]. Despite advancements in imaging and surgical techniques, TAAD continues to result in high rates of morbidity and mortality. The disease often progresses silently, making early detection difficult and leading to late-stage presentations and frequent misdiagnoses [5,6]. Current clinical management primarily focuses on controlling blood pressure using β-blockers or angiotensin II type 1 receptor blockers, along with elective surgical repair once aneurysms reach certain size thresholds [7]. However, no pharmacological treatment has been conclusively shown to halt or reverse the progression of aneurysms [8,9,10,11]. Therefore, a deeper understanding of the mechanisms behind aortic wall degeneration is crucial for identifying new therapeutic strategies.

A distinct pathological feature of TAAD is medial degeneration, characterized by the loss of vascular smooth muscle cells (VSMCs), fragmentation of elastic fibers, and remodeling of the extracellular matrix (ECM). VSMCs are the primary cell type in the aortic wall, and they play a crucial role in maintaining vascular integrity by synthesizing elastin, collagen, and other matrix components. Additionally, they perform a contractile function that is essential for distributing mechanical stress [1,12,13,14]. Multiple forms of regulated cell death have been linked to the development of thoracic aortic aneurysms, including apoptosis [15,16], necroptosis [17,18], pyroptosis [19,20], and autophagy-related cell death [21,22,23]. Recently, ferroptosis—a regulated form of cell death that is iron-dependent and driven by the accumulation of lipid-based reactive oxygen species (ROS) and a failure of antioxidant defense systems—has gained significant interest in the study of aortic pathology [24,25,26,27].

In vascular biology, ferroptosis has been shown to worsen endothelial dysfunction [28,29] and inflammation [30,31]. VSMCs, when triggered by iron overload, ROS accumulation, and subsequent lipid peroxidation, undergo a detrimental ferroptosis-induced phenotypic switch, including transformation toward an osteogenic phenotype [32,33]. Additionally, ferroptotic signaling accelerates arterial stiffening by promoting VSMC senescence through NCOA4-mediated ferritinophagy [34]. Interestingly, in cases of aortic dissection, oxidative stress-induced activation of the HIF-1α/HO-1 signaling pathway leads to heme-derived iron accumulation. This process results in VSMC ferroptosis, which ultimately triggers significant degeneration of the arterial media and compromises the integrity of the aortic wall [35]. While targeting ferroptosis represents a promising therapeutic strategy for TAAD [36,37], further research is needed to clarify the complex regulatory networks involved and to assess the effectiveness of novel ferroptosis inhibitors.

Ketone bodies, primarily β-hydroxybutyrate (β-OHB), acetoacetate, and acetone, are endogenous metabolites produced by the liver’s mitochondria during periods of low carbohydrate availability, such as fasting or following a ketogenic diet [38]. During metabolic stress, such as heart failure, diabetic cardiomyopathy, and ischemia/reperfusion, ketone bodies serve as highly efficient rescue fuels that helps restore energetic homeostasis [39,40,41]. In addition to functioning as alternative energy sources, ketone bodies have various signaling effects. In the heart and blood vessels, β-OHB has been shown to enhance mitochondrial efficiency, reduce oxidative stress, modulate inflammatory signaling pathways, improve endothelial function, and restore VSMC contractility [38,42,43,44,45,46,47,48]. Mechanistically, β-OHB acts as a signaling molecule that can inhibit histone deacetylases (HDACs), activate G-protein coupled receptors such as HCAR2, suppress NLRP3 inflammasome activation, and directly modify histones through lysine β-hydroxybutyrylation [49,50,51,52]. While recent research has begun to explore the therapeutic potential of β-OHB in aortic diseases such as abdominal aortic aneurysm [53], its effect on aortic medial degeneration in TAAD remains unclear. This study aims to explore the hypothesis that ketone ester supplementation can reduce TAAD by suppressing ferroptosis in VSMCs. By integrating in vivo and mechanistic analyses, we seek to determine whether targeting ferroptotic pathways through ketone ester-mediated signaling can decrease medial degeneration, limit ECM breakdown, and ultimately slow the progression of TAAD.

## 2. Materials and Methods

### 2.1. Animal Studies

All animal procedures were conducted in strict accordance with the guidelines approved by the Georgia State University (GSU) Institutional Animal Care and Use Committee (IACUC). C57BL/6 wild-type mice were housed in a climate-controlled facility (21 ± 2 °C) under a 12:12 h light–dark cycle, with ad libitum access to standard chow and water. To induce TAAD, 3-week-old male and female mice were given β-aminopropionitrile (BAPN) monofumarate (Sigma-Aldrich, St. Louis, MO, USA, Cat. No. A3134) through their drinking water at a dosage of 1.2 g/kg/day for 28 days. BAPN acts as an irreversible inhibitor of lysyl oxidase, which prevents the cross-linking of elastin and collagen fibers, thereby compromising the integrity of aortic media [54,55]. For the treatment group, mice were supplemented with (R)-3-hydroxybutyl (R)-3-hydroxybutyrate ketone ester (KE) (KetoneAid, Falls Church, VA, USA) at a concentration of 20 g/L in their drinking water, starting on day 15 of the BAPN protocol and continuing for the remaining 14 days. The final experimental groups consisted of 7 male and 7 female control mice, 16 male and 13 female BAPN mice, and 25 male and 12 female mice that received both BAPN and KE.

Animal health, activity, and survival were monitored daily. Dosage was adjusted based on the average water consumption and body weight of the mice, with body weight recorded every 48 h. Systemic blood ketone levels were measured through tail vein sampling on day 14 (before KE) and at multiple intervals throughout the KE supplementation period. Mice that experienced aortic rupture before the 28-day endpoint were immediately autopsied to confirm TAAD-related mortality. On day 28, the surviving mice were euthanized by CO_2_ inhalation followed by cervical dislocation, in accordance with GSU IACUC policy. The aortas were then harvested. Aortas were either fixed in 10% neutral buffered formalin for 24 h or snap-frozen in liquid nitrogen and stored at −80 °C for subsequent histological, protein, and gene expression analyses.

### 2.2. Histological Staining

Thoracic aortic tissue samples were fixed in formalin, embedded in paraffin, and sectioned to a thickness of 5 µm. For structural and pathological evaluation, the formalin-fixed paraffin-embedded slides underwent several staining procedures: Hematoxylin and Eosin (H&E) (Sigma-Aldrich, St. Louis, MO, USA, Cat. No. HHS32/HT110132) to assess general morphology and medial thickness; Masson’s Trichrome (Sigma-Aldrich, St. Louis, MO, USA, Cat. No. HT15) to visualize collagen deposition and interstitial fibrosis; Verhoeff–Van Gieson (VVG) (Newcomer Supply, Waunakee, WI, USA, Cat. No. 9116A) to evaluate the integrity of the elastic lamellae; and Prussian Blue (Gomori method) (Newcomer Supply, Waunakee, WI, USA, Cat. No. 9136A) to detect ferric iron (Fe^3+^) deposits. Quantitative morphometric analysis was performed using Fiji/ImageJ 1.54g (Wayne Rasband, National Institutes of Health, Bethesda, MD, USA) [56]. The arterial medial thickness was calculated from four equidistant points along the circumference of H&E-stained aortic sections. Collagen deposition was quantified as a percentage of the total area, utilizing the Color Deconvolution plugin to isolate the methyl blue-stained fibers. The number of elastin breaks was determined by manually counting discontinuities in the elastic laminae across the entire aortic cross-section in the VVG-stained slides. Finally, iron accumulation was quantified by calculating the density of Prussian blue-positive spots (spots/mm^2^) through manual counting.

### 2.3. Immunohistochemistry (IHC) Staining

For immunohistochemical evaluation, 5 µm paraffin sections were deparaffinized in xylene and rehydrated through a graded series of ethanol. Heat-induced antigen retrieval was performed using sodium citrate buffer (pH 6.0). To minimize non-specific background staining, endogenous peroxidase activity was quenched with 3% hydrogen peroxide for 10 min, followed by a blocking step with 5% normal goat serum (BioGenex, Fremont, CA, USA, Cat. No. HK112-9K) for one hour at room temperature. Sections were then incubated overnight at 4 °C with the indicated primary antibodies (Supplementary Materials, Table S1) and subsequently for 1 h with secondary antibodies following washing. Signals were visualized using the Liquid DAB+ Substrate Chromogen System (Dako North America, Inc., Carpinteria, CA, USA, Cat. No. K3468), counterstained with hematoxylin, dehydrated, and mounted. Images were captured using an Olympus microscope (BX53TF, Tokyo, Japan). Quantitative analysis was performed using ImageJ/Fiji software.

### 2.4. Cell Culture, Treatments, and siRNA Transfection

Primary human aortic smooth muscle cells (HASMCs) (Lonza, Walkersville, MD, USA, Cat. No. CC-2571) were maintained in specialized SmGM-2 growth medium (Lonza, Walkersville, MD, USA, Cat. No. CC-3182) at 37 °C in a humidified 5% CO_2_ atmosphere. For signaling and rescue experiments, HASMCs were treated with β-OHB (5 mM for 24 h), the ferroptosis inhibitor Ferrostatin-1 (2 µM for 24 h), or the GPX4 inhibitor RSL3 (50 nM for 24 h or 200 nM for 2 h). To achieve targeted gene silencing, HASMCs were transfected with specific small interfering RNAs (siRNAs) against *GPX4* (Assay ID 10848) or *NFE2L2* (Assay ID 107966) using Lipofectamine RNAiMAX (Invitrogen, Waltham, MA, USA, Cat. No. 13778150) according to the manufacturer’s lipid-mediated delivery protocol.

### 2.5. Western Blotting

Total protein was extracted from either cleared murine aortic tissues or HASMC lysates using RIPA lysis buffer (Santa Cruz, Dallas, TX, USA, Cat. No. sc-24948A) supplemented with protease and phosphatase inhibitors. Protein concentrations were determined using the Bicinchoninic Acid (BCA) Protein Assay Kit (Thermo Fisher Scientific, Waltham, MA, USA, Cat. No. 23225). Samples were resolved via SDS-PAGE and then transferred onto polyvinylidene difluoride (PVDF) membranes (Millipore Sigma, Burlington, MA, USA, Cat. No. IPVH00005). The membranes were blocked with 5% non-fat dry milk and subsequently probed with primary and then secondary antibodies (Supplementary Materials Table S1) after a wash step. Protein bands were visualized using enhanced chemiluminescence reagent (Thermo Fisher Scientific, Waltham, MA, USA, Cat. No. 32134) and captured using the Amersham Imager 680 (GE Healthcare, Uppsala, Sweden). Different loading controls (β-actin, GAPDH, and β-tubulin) were used across various experiments, allowing for the simultaneous probing of multiple target proteins with differing molecular weights on the same membrane. Densitometry quantification was performed using ImageJ/Fiji software, with target protein signals normalized to their respective loading controls.

### 2.6. RNA Isolation and Real-Time PCR (RT-qPCR)

Total RNA was isolated from cleared murine aortic tissues or HASMCs using Trizol Reagent (Invitrogen, Waltham, MA, USA, Cat. No. 15596026), following the manufacturer’s instructions. The concentration and purity of the RNA were assessed using a NanoDrop spectrophotometer (Thermo Fisher Scientific, Waltham, MA, USA). Complementary DNA (cDNA) was synthesized from 1 µg of total RNA using the iScript cDNA Synthesis Kit (Bio-Rad, Hercules, CA, USA, Cat. No 1708890). Quantitative real-time PCR (RT-qPCR) was conducted using 2X SYBR Green qPCR Master Mix (Selleckchem, Houston, TX, USA, Cat. No. B21203) on a Bio-Rad CFX96 Real-Time PCR Detection System. Relative mRNA expression levels were calculated using the 2^−ΔΔCT^ method and normalized to the internal housekeeping genes *GAPDH* or *ACTB*. The specific primer sequences used for RT-qPCR are detailed in Supplementary Materials Table S2.

### 2.7. Lipid Peroxidation Assay

To evaluate lipid peroxidation, HASMCs underwent specific treatments and were then incubated with 5 µM C11-BODIPY 581/591 (Thermo Fisher Scientific, Waltham, MA, USA, Cat. No. D3861) for 30 min at 37 °C. C11-BODIPY is a lipid-sensitive fluorophore that shifts its emission maximum from red (approximately 590 nm) to green (approximately 510 nm) upon oxidation. Fluorescence images were captured using a fluorescence microscope, and the extent of lipid peroxidation was quantified by calculating the ratio of green (oxidized) to total fluorescence intensity (oxidized + reduced) using ImageJ/Fiji.

### 2.8. Malondialdehyde Quantification

The concentration of malondialdehyde (MDA), a secondary byproduct of lipid peroxidation, was measured in lysates of HASMCs that had undergone specific cell treatments. This measurement was performed using the Thiobarbituric Acid Reactive Substances (TBARS) Assay Kit (Cayman Chemical, Ann Arbor, MI, USA, Cat. No. 10009055). In this assay, samples and standards were reacted with thiobarbituric acid at high temperature to produce a colorimetric product. The absorbance of this product was then measured at a wavelength of 532 nm using a microplate reader.

### 2.9. Intracellular Iron Detection

Intracellular labile ferrous iron (Fe^2+^) was detected using the live-cell fluorescent probe FerroOrange (Dojindo, Rockville, MD, USA, Cat. No. F374). HASMCs subjected to the specified treatments were washed with serum-free medium and incubated with 1 µM FerroOrange at 37 °C for 30 min. After incubation, the red fluorescence intensity, which specifically increases upon reaction with Fe^2+^, was visualized and captured using fluorescence microscopy. Quantitative analysis of the mean fluorescence intensity was performed using ImageJ/Fiji software.

### 2.10. Bioinformatic Analyses

Publicly available GEO datasets were analyzed using R (version 4.5.2) to evaluate the expression of ferroptosis-related genes in human aortic disease. Microarray data from GSE52093 (5 normal vs. 7 STAAD samples) [57] and high-throughput sequencing data from GSE153434 (10 normal vs. 10 TAAD samples) [58] were used to identify differentially expressed genes (DEGs), with a specific focus on the expression profiles of key ferroptosis markers, including *GPX4*, *HMOX1*, *TFRC*, and *FTH1*. For single-cell analysis, the GSE155468 dataset [59] was employed to perform Gene Set Enrichment Analysis (GSEA) on clusters of vascular smooth muscle cells (SMCs). The enrichment of “Ferroptosis Suppressors” was compared between human control samples and samples from patients with Acute Thoracic Aortic Aneurysm (ATAA) to determine cell-type-specific metabolic shifts.

### 2.11. Statistical Analysis

Data are expressed as mean ± standard error of the mean (SEM) as indicated in the figure legends. Statistical comparisons between two groups were performed using a two-tailed Student’s *t*-test. For comparisons involving three or more groups, one-way ANOVA followed by Tukey’s post hoc test was employed. To analyze experiments with two independent variables, two-way ANOVA followed by Sidak’s or Tukey’s multiple comparisons test was utilized. Survival curves were analyzed using the Log-rank (Mantel–Cox) test, and the incidence graph was analyzed using the two-tailed Fisher’s exact test. A *p*-value < 0.05 was considered statistically significant. All statistical analyses and graphing were performed using GraphPad Prism 8.0 software (GraphPad Software, San Diego, CA, USA).

## 3. Results

### 3.1. Ketone Ester Reduces the Incidence of BAPN-Induced TAAD

**Figure 1:**
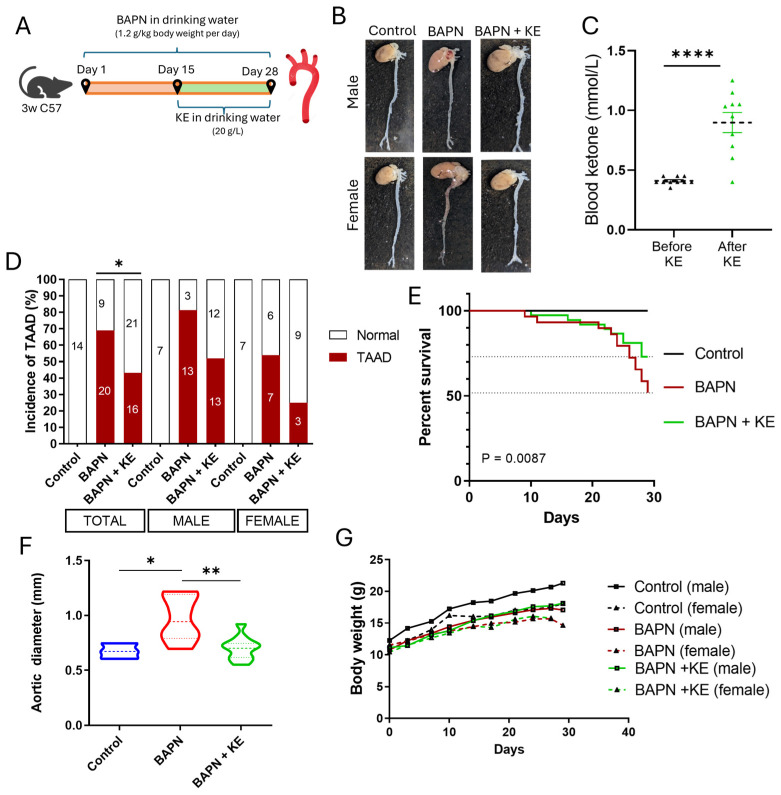
Ketone ester protects C57BL/6 mice from BAPN-induced TAAD. Three-week-old C57BL/6 mice were given BAPN (1.2 g/kg/day) in their drinking water for 28 days. One treatment group was supplemented with ketone ester (KE, 20 g/L) in their drinking water. (**A**) The experimental timeline and workflow for the administration of BAPN and KE are outlined. (**B**) Representative macroscopic images of the aorta from male and female mice in the Control, BAPN, and BAPN + KE groups are shown. (**C**) Violin plots illustrate systemic blood ketone concentrations before and after KE supplementation. (**D**) The incidence rate of TAAD across the experimental cohorts is presented. (**E**) Kaplan–Meier survival analysis demonstrates the protective effect of KE against BAPN-induced lethality. The Log-rank (Mantel–Cox) test used for statistical analysis. (**F**) Quantification of maximal thoracic aortic diameter (in mm) at the 28-day endpoint. (**G**) Longitudinal body weight measurements throughout the 4-week experimental period. Data are presented as mean ± SEM, *n* = 4–8, * *p* < 0.05, ** *p* < 0.01, **** *p* < 0.0001.

To investigate the potential protective effects of exogenous ketosis on vascular health, we used a well-established mouse model of TAAD, administering BAPN to 3-week-old C57BL/6 mice (Figure 1A). Systematic administration of KE in the drinking water successfully induced a state of nutritional ketosis, as indicated by a significant increase in systemic blood ketone levels, rising from a baseline of 0.3 mmol/L to 0.9 mmol/L following supplementation (Figure 1C). Notably, blood ketone levels peaked at night, likely due to increased fluid consumption by the mice during the nighttime. Macroscopic examination of the harvested aorta revealed distinct morphological differences among the experimental groups. Mice challenged with BAPN showed pronounced aneurysmal dilation, particularly in the ascending thoracic aorta region. Remarkably, KE supplementation visibly reduced these pathological changes in both male and female mice (Figure 1B). The protective effects of KE were further demonstrated by the incidence of TAAD. In the BAPN-only group, a high incidence of TAAD was observed (69%). In contrast, KE supplementation significantly reduced this rate to 43% compared to the BAPN group (Figure 1D). The trend observed in the study was consistent across both sexes. Male mice showed a reduction in TAAD development from 81% to 52% following KE treatment, while female mice exhibited a decrease from 54% to 25%. This indicates a higher incidence rate of TAAD in male mice compared to female mice; however, the relative protective effects of KE supplementation are conserved in both male and female mice.

Kaplan–Meier survival analysis revealed that KE supplementation significantly improved the survival rates of mice experiencing lethality induced by BAPN, increasing survival from 52% to 73% (Figure 1E). Quantification of the maximal thoracic aortic diameter at the 28-day endpoint showed similar findings. BAPN administration resulted in a significant increase in mean external aortic diameter compared to Controls (0.9678 mm vs. 0.6743 mm). In contrast, KE treatment effectively mitigated this dilation, resulting in a mean diameter of 0.7015 mm (Figure 1F). Finally, longitudinal body weight measurements indicated consistent growth across all cohorts, with no significant differences between the groups (Figure 1G).

### 3.2. Ketone Ester Preserves Aortic Structural Integrity and Prevents Pathological Remodeling

**Figure 2:**
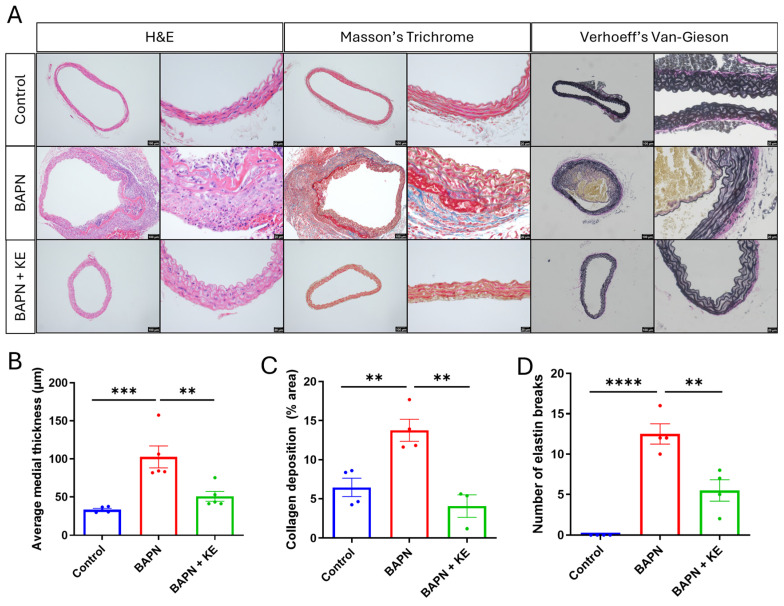
Ketone ester helps maintain the structural integrity of the aorta in BAPN-induced TAAD. (**A**) Representative histological images of the thoracic aorta stained with Hematoxylin and Eosin (H&E; showing blue/purple nuclei and pink cytoplasm/extracellular matrix), Masson’s Trichrome (highlighting blue collagen fibers and red muscle fibers), and Verhoeff–Van Gieson (VVG; identifying black elastic fibers and red collagen/muscle) stains. Scale bars = 100 µm and 20 µm. (**B**) Quantification of average medial thickness (in µm) across the experimental groups. (**C**) Quantitative analysis of collagen deposition, expressed as the percentage of area positive for Masson’s Trichrome staining. (**D**) Quantification of the number of elastic fiber breaks per high-power field as assessed by VVG staining. Data are presented as mean ± SEM (*n* = 4 for each group). ** *p* < 0.01, *** *p* < 0.001, **** *p* < 0.0001.

We conducted a thorough histological assessment of the thoracic aorta to evaluate the impact of KE on the structural disintegration associated with TAAD. As shown in Figure 2A, H&E staining revealed a significant expansion of the medial layer in the BAPN-treated group compared to the control group. Notably, this increase in average medial thickness was significantly reduced in mice that received KE supplementation (Figure 2A,B). To further analyze the composition of collagen and elastin, we employed Masson’s Trichrome and Verhoeff–Van Gieson staining techniques. The administration of BAPN led to a marked increase in collagen deposition, indicative of reactive fibrosis (Figure 2A,C). Conversely, the BAPN + KE group exhibited significantly lower levels of collagen accumulation, more closely resembling the profile of the control group. Crucially, VVG staining showed that while the control aortas had intact, continuous elastic lamellae, the BAPN group exhibited extensive fragmentation of elastic fibers, which is a key histopathological indicator of TAAD [60]. The number of elastin breaks per high-power field was significantly higher in the BAPN-treated mice; however, KE treatment substantially reduced the occurrence of these breaks (Figure 2D). Collectively, these findings indicate that KE supplementation helps preserve the structural integrity of the aortic wall.

### 3.3. Ferroptosis Is Associated with TAAD in Both Humans and Mice

**Figure 3:**
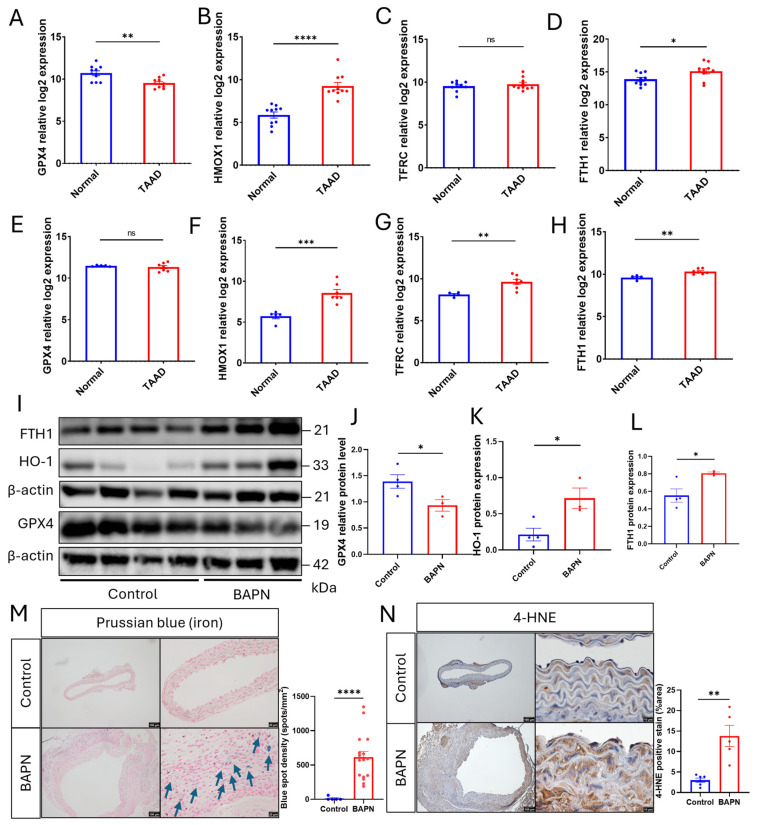
Ferroptosis is associated with both human and murine TAAD. (**A**–**D**) Relative log2 mRNA expression levels of ferroptosis-related genes (*GPX4*, *HMOX1*, *TFRC*, and *FTH1*) in human normal aorta vs. Stanford Type A Aortic Dissection (STAAD) samples from the GSE153434 dataset. (**E**–**H**) Validation of ferroptosis gene expression (*GPX4*, *HMOX1*, *TFRC*, and *FTH1*) in a second independent human cohort (GSE52093). (**I**) Representative Western blot images of FTH1, HO-1, and GPX4 protein levels in whole aortic tissue from Control and BAPN-treated mice. (**J**–**L**) Densitometric quantification of GPX4, HO-1, and FTH1 protein expression normalized to β-actin. (**M**) Representative images of Prussian blue staining (blue arrows indicating iron spots) in Control and BAPN-treated aortas (scale bars = 100 µm and 20 µm) and corresponding quantification of blue spot density (spots/mm^2^). (**N**) Immunohistochemical staining for 4-Hydroxynonenal (4-HNE) in both Control and BAPN-treated groups (scale bars = 100 µm and 10 µm) with quantitative analysis of 4-HNE-positive area (%). Data are presented as mean ± SEM (*n* = 4–16 per group). * *p* < 0.05, ** *p* < 0.01, *** *p* < 0.001, **** *p* < 0.0001, ns *p* > 0.05.

To explore the clinical significance of ferroptosis in aortic conditions like TAAD, we first analyzed publicly available transcriptomic datasets from human patients. In the GSE153434 dataset, Stanford Type A Aortic Dissection (STAAD) samples exhibited a significant downregulation of *GPX4*, a key ferroptosis suppressor, coupled with a robust induction of the pro-ferroptotic and iron-handling genes *HMOX1* and *FTH1* (Figure 3A–D). These results were largely validated in a second independent human STAAD cohort (GSE52093), which consistently showed significant elevations in *HMOX1*, *TFRC*, and *FTH1* in dissected aortas compared to normal aortas (Figure 3E–H). FTH1, an essential component of ferritin, plays an anti-ferroptotic role by sequestering free ferrous ions [61]. The elevated levels of *FTH1* and *TFRC* in TAAD may indicate a heightened demand for iron sequestration due to iron accumulation, facilitated by iron importers such as Transferrin receptor 1.

The SMC-specific Gene Set Enrichment Analysis (GSEA) of scRNA-seq data from human control and aortic tissue aneurysm samples (ATAA) samples (GSE155468) revealed a significant negative enrichment of the “Ferroptosis Suppressor” gene set in the aortic tissue aneurysm samples (Supplementary Figure S1A). The enrichment plot demonstrates a clear downward shift in the running enrichment score, indicating that most genes responsible for preventing lipid peroxidation and ferroptotic cell death are markedly downregulated in the SMCs of diseased human aortas.

We next aimed to determine whether the ferroptotic signatures were present in our BAPN-treated mouse model. Western blot analysis of whole aortic lysates validated that the protein expression of GPX4 was significantly decreased in the BAPN group, while levels of HO-1 and FTH1 were significantly increased (Figure 3I–L), consistent with the human data. Further functional evidence of ferroptosis was provided by Prussian blue staining, which revealed a significant accumulation of iron deposits in the aortas of BAPN-treated mice, particularly in areas with marked medial thickening. In contrast, iron was almost undetectable in control aortic tissues (Figure 3M). Additionally, immunohistochemical staining for 4-HNE, a definitive marker of lipid peroxidation [62], showed a significantly larger positive area in the BAPN group compared to the controls (Figure 3N). Together, these findings from human datasets and our murine model provide strong evidence that ferroptosis is a key pathological feature associated with the progression of TAAD.

### 3.4. Ketone Ester and β-OHB Mitigate Lipid Peroxidation and Iron Accumulation

**Figure 4:**
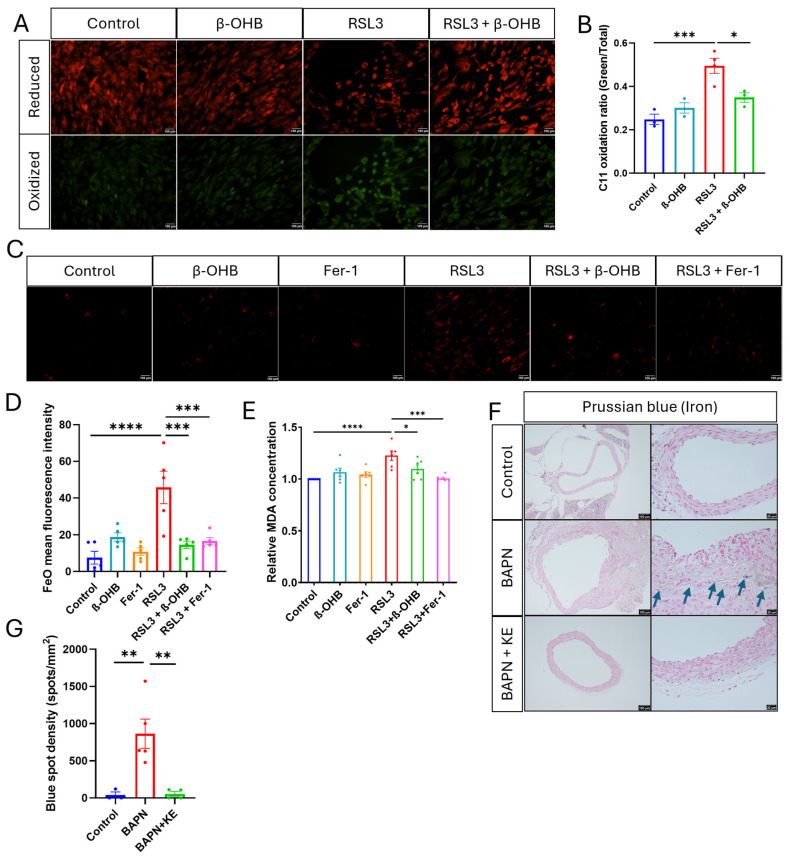
Ketone ester and β-OHB mitigate lipid peroxidation and iron accumulation. (**A**) Representative fluorescence images of C11-BODIPY staining in HASMCs from various treatment groups: Control, β-OHB, RSL3, and RSL3 + β-OHB treatment groups, showing both reduced (red) and oxidized (green) lipid signals. Scale bar = 100 µm. (**B**) Quantitative analysis of the C11-BODIPY fluorescence ratio (Oxidized/Total), indicating the degree of lipid peroxidation. (**C**) Representative fluorescence images of FerroOrange staining in HASMCs used to detect intracellular labile iron (Fe^2+^) in Control, β-OHB, Fer-1, RSL3, RSL3 + β-OHB, and RSL3 + Fer-1 groups. Scale bar = 100 µm. (**D**) Quantification of FerroOrange Mean Fluorescence Intensity. (**E**) Relative concentration of malondialdehyde (MDA) measured via TBARS assay across the indicated experimental groups in HASMCs. (**F**) Representative images of Prussian blue staining in aortic tissue sections from Control, BAPN, and BAPN + KE mice cohortsblue arrows indicating in vivo iron deposition. Scale bars = 100 µm and 20 µm. (**G**) Quantification of Prussian blue spot density (spots/mm^2^). Data are presented as mean ± SEM (*n* = 3–6 per group). * *p* < 0.05, ** *p* < 0.01, *** *p* < 0.001, **** *p* < 0.0001. β-OHB: 5 mM; Fer-1 (Ferrostatin-1): 2 µM; RSL3: 200 nM (2 h).

To investigate the role of β-OHB in preventing ferroptosis, we evaluated lipid peroxidation and iron homeostasis in HASMCs. Using the lipid-sensitive probe C11-BODIPY, we found that the GPX4 inhibitor RSL3 induced a significant shift from red (indicating reduced levels) to green (indicating oxidized levels) fluorescence, which is a hallmark of ferroptosis (Figure 4A). Importantly, co-treatment with β-OHB significantly lowered the oxidized/total lipid ratio (Figure 4B), indicating its strong antioxidant properties. This finding was further supported by the TBARS assay, which showed that β-OHB effectively reduced the RSL3-induced increase in malondialdehyde (MDA) levels (Figure 4E), a stable byproduct of lipid peroxidation.

Given that labile iron (Fe^2+^) is the main driver of the Fenton reaction in ferroptosis [63], we used the FerroOrange probe to detect intracellular iron levels. Treatment with RSL3 led to a significant increase in mean fluorescence intensity (MFI), indicating an accumulation of pro-ferroptotic labile iron (Figure 4C,D). Interestingly, treatment with β-OHB or the ferroptosis inhibitor Ferrostatin-1 (Fer-1) significantly reduced the labile iron pool, suppressing the RSL3-induced iron overload. Furthermore, in vivo Prussian blue staining on murine aortic sections also validated these findings. Mice treated with BAPN showed high densities of iron deposits in the aortic media, while the BAPN + KE group exhibited a near-complete absence of these deposits, similar to the control group (Figure 4F,G). These results suggest that ketone supplementation effectively limits iron accumulation and lipid peroxidation.

### 3.5. Ketone Ester Supplementation Restores the Antioxidant GPX4/SLC7A11 Axis and Suppresses Ferroptotic Stress In Vivo

**Figure 5:**
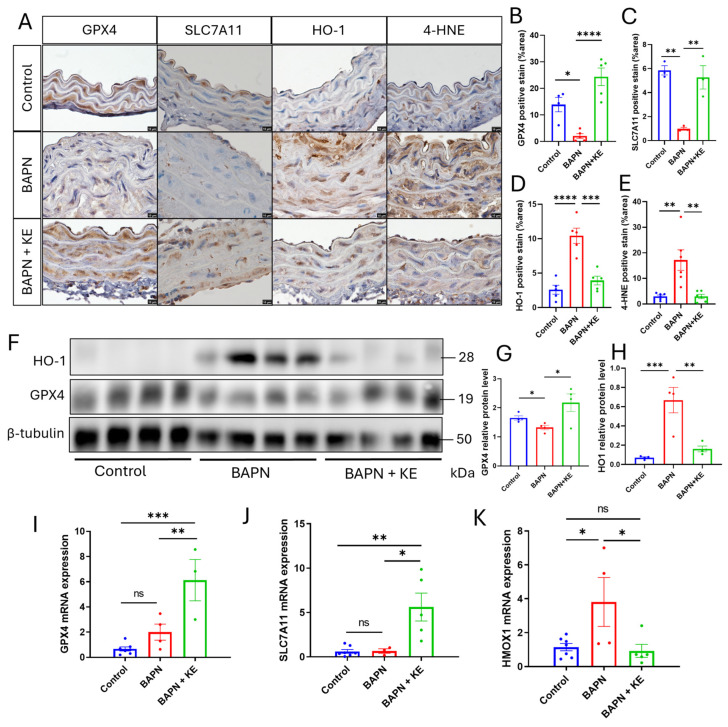
Ketone ester supplementation prevents ferroptosis in BAPN-induced TAAD. (**A**) Representative IHC images showing the expression and localization of GPX4, SLC7A11, HO-1, and 4-HNE in the aortic media of Control, BAPN, and BAPN + KE mice groups. Scale bar = 10 µm. (**B**–**E**) Quantitative analysis of IHC staining for (**B**) GPX4, (**C**) SLC7A11, (**D**) HO-1, and (**E**) 4-HNE, expressed as the percentage of positive staining area (%). (**F**) Representative Western blot images of GPX4, SLC7A11, and HO-1 protein levels from whole aortic lysates across the indicated experimental groups. (**G**,**H**) Densitometric quantification of protein expression normalized to the β-tubulin loading control. (**I**–**K**) Relative mRNA expression levels of (**I**) *GPX4*, (**J**) *SLC7A11*, and (**K**) *HMOX1* in thoracic aortic tissues, as determined by RT-qPCR. Data are presented as mean ± SEM (*n* = 4–6 per group). * *p* < 0.05, ** *p* < 0.01, *** *p* < 0.001, **** *p* < 0.0001, ns *p* > 0.05.

To determine how KE prevents aortic degeneration, we examined the expression of key ferroptosis regulators and lipid peroxidation markers in the murine aorta. IHC analysis revealed that BAPN treatment significantly reduced the levels of GPX4 and SLC7A11 in the aortic media (Figure 5A–C). Remarkably, KE supplementation significantly increased the expression of these antioxidant proteins compared to the BAPN group. In contrast, the oxidative stress marker 4-HNE and the stress-inducible HO-1 were markedly elevated in BAPN-treated aortas, both of which were significantly reduced by KE treatment (Figure 5D,E). These findings were further validated at the protein level using Western blot analysis of whole aortic lysates. Consistent with the IHC results, the BAPN group exhibited a significant reduction in GPX4 protein expression along with a substantial elevation of HO-1 (Figure 5F–H). Supplementation with KE successfully restored GPX4 levels and suppressed the pathological increase in HO-1.

To investigate whether these changes were driven at the transcriptional level, we performed RT-qPCR on thoracic aortic tissues. While BAPN treatment alone did not significantly alter the mRNA levels of *GPX4* and *SLC7A11* compared to controls, the addition of KE significantly upregulated the transcription of both genes (Figure 5I,J). Furthermore, the BAPN-induced increase in *HMOX1* mRNA was significantly reversed by KE (Figure 5K). Collectively, these results imply that KE protects the aorta, likely via transcriptionally upregulating the System Xc^−^/GPX4 antioxidant defense system and mitigating the iron-related stress response characterized by HO-1 overexpression.

### 3.6. β-OHB Prevents Ferroptosis by Antagonistically Regulating the GPX4 and HO-1 Pathways Through Nrf2

**Figure 6:**
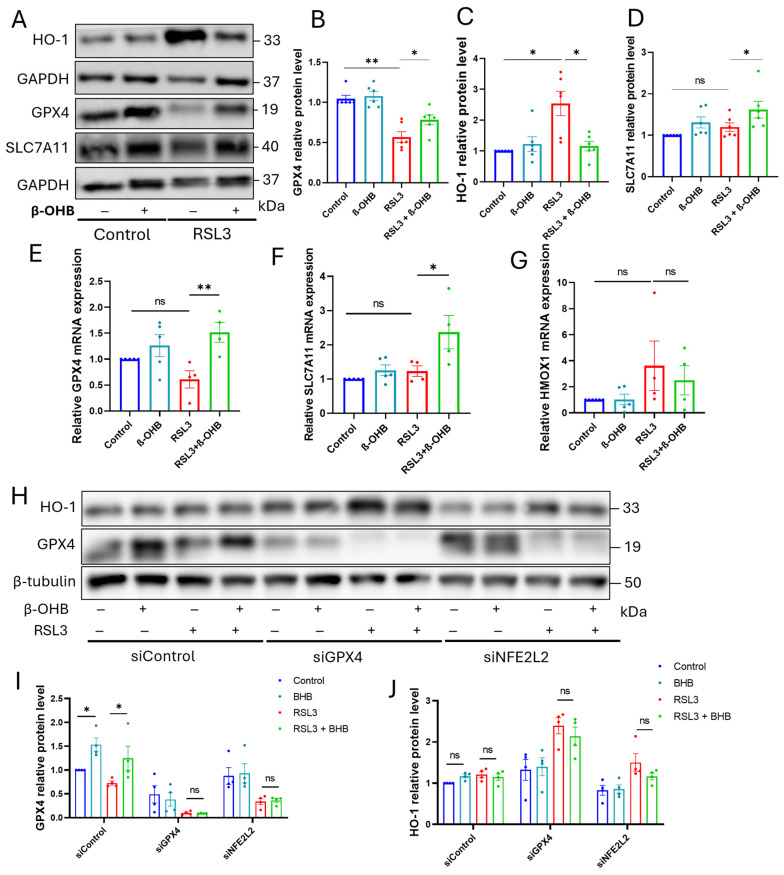
β-OHB prevents ferroptosis by antagonistically regulating the GPX4 and HO-1 pathways. (**A**) Representative Western blot images for HO-1, GPX4, and SLC7A11 in VSMCs treated with β-OHB and/or the GPX4 inhibitor RSL3. (**B**–**D**) Densitometric quantification of (**B**) GPX4, (**C**) HO-1, and (**D**) SLC7A11 protein expression levels normalized to the GAPDH loading control. (**E**–**G**) Relative mRNA expression of (**E**) *GPX4*, (**F**) *SLC7A11*, and (**G**) *HMOX1* in HASMCs across the indicated treatment groups, as measured by RT-qPCR. (**H**) Representative Western blot analysis of GPX4 and HO-1 protein levels in HASMCs transfected with control siRNA (siControl), *GPX4*-targeted siRNA (siGPX4), or *NFE2L2*-targeted siRNA (siNFE2L2) and subsequently treated with RSL3 and/or β-OHB. (**I**,**J**) Quantitative densitometric analysis of the protein expression levels shown in Panel (**H**). Data are presented as mean ± SEM (*n* = 4–6 per group). * *p* < 0.05, ** *p* < 0.01, ns *p* > 0.05. β-OHB: 5 mM; RSL3: 50 nM (24 h).

To elucidate the molecular mechanism behind the protective effects of β-OHB, we conducted pharmacological inhibition and gene silencing experiments in HASMCs. Western blot analysis showed that the GPX4 inhibitor RSL3 significantly reduced GPX4 protein levels while causing a compensatory increase in the stress-responsive enzyme HO-1. Co-treatment with β-OHB effectively restored GPX4 expression and suppressed the RSL3-induced rise in HO-1 (Figure 6A–C). Although RSL3 treatment led to a depletion of GPX4 levels, dose-dependent treatment with β-OHB resulted in a progressive increase in GPX4 levels, both under baseline conditions and when treated with RSL3 (Supplementary Figure S1C).

Additionally, while RSL3 did not significantly alter SLC7A11 levels, β-OHB treatment substantially increased SLC7A11 expression at both the protein and mRNA levels (Figure 6D,F), thereby enhancing the System Xc^−^ antioxidant pathway. To determine if this regulation occurs at the transcriptional level, we performed RT-qPCR. Although RSL3 alone did not significantly deplete *GPX4* mRNA, the addition of β-OHB led to a significant transcriptional upregulation of both *GPX4* and *SLC7A11* (Figure 6E,F). Interestingly, the increase in *HMOX1* mRNA induced by RSL3 was moderately reduced by β-OHB (Figure 6G), suggesting a shift in the cellular response from an iron-releasing state toward a more stable antioxidant defense.

We investigated the role of the master antioxidant transcription factor Nrf2 (*NFE2L2*) and its primary target, GPX4, in the mechanism we studied. In cells that were transfected with control siRNA, β-OHB consistently increased GPX4 expression under both baseline conditions and when challenged with RSL3 (Figure 6H–J). However, in cells where *NFE2L2* was silenced (siNFE2L2), the ability of β-OHB to reverse RSL3-induced GPX4 depletion was completely lost. Similarly, in the siGPX4 group, RSL3 treatment led to an increased HO-1 stress response, which β-OHB was less effective at alleviating. These results suggest that β-OHB prevents ferroptosis by activating an Nrf2-dependent transcriptional program that sustains the GPX4/SLC7A11 axis while counteracting the pathological HO-1 stress response.

## 4. Discussion

The present study demonstrates that supplementing with exogenous ketones, particularly through the use of KE, significantly protects against the development and progression of TAAD in mice. By employing a BAPN-induced mouse model and conducting human transcriptomic analyses along with in vitro assays on HASMCs, we identified ferroptosis as one of the key pathological factors contributing to aortic medial degeneration. Our findings also demonstrate that β-OHB helps counteract this process by stabilizing the antioxidant system, likely through an Nrf2-dependent transcriptional program. Although the antioxidant properties of β-OHB are acknowledged in many areas, TAAD represents a unique vascular pathology. This study provides novel evidence that the β-OHB–Nrf2–GPX4 axis specifically prevents the ferroptotic breakdown of VSMCs. This mechanism helps maintain the integrity of the thoracic aortic media and inhibits the progression of TAAD.

Ferroptosis is a key characteristic of TAAD, as demonstrated in this study and others [35,36,37]. Existing literature supports that ferroptosis is a causal driver of TAAD. For example, studies using a BAPN-induced model have shown that inhibition of ferroptosis with liproxstatin-1 significantly reduces the incidence of aortic dissection [37]. Similar protective effects have been observed with ferrostatin-1 in abdominal aortic aneurysm models [64], thus establishing a causal link between ferroptosis and aortic pathology. Our analysis of multiple human cohorts (GSE52093, GSE153434, and GSE155468) consistently revealed an association between ferroptosis and diseased tissues. This association is characterized by the downregulation of antioxidant ferroptosis suppressors, specifically *GPX4*, and the upregulation of iron-handling molecules such as *TFRC*, *FTH1*, and *HMOX1*. These findings were further validated through scRNA-seq gene set enrichment analysis (GSEA) for the ferroptosis suppressor gene set, which highlighted these changes specifically within VSMC clusters. FTH1, as previously mentioned, is crucial for sequestering free ferrous iron and converting it into ferric iron for storage [61]. Although FTH1 is anti-ferroptotic, an increase in its expression in this context should be interpreted as a heightened cellular response to iron overload, a compensatory mechanism aimed at sequestering excess iron.

In our BAPN mouse model, we observed significant iron accumulation, as indicated by Prussian blue staining, particularly in areas with medial thickening. This spatial correlation suggests that iron-induced oxidative stress is concentrated in the dilated segments of the aorta. This condition may serve as a primary trigger for VSMC dysfunction, leading to subsequent structural failures that culminate in aortic dissection.

A particularly striking finding from this study is the apparent antagonism between GPX4 and HO-1 during the progression of TAAD. While GPX4, the primary enzyme responsible for neutralizing lipid peroxides and a key player in preventing ferroptosis, is reduced in diseased aortas, HO-1 is dramatically upregulated. Our gene silencing experiments demonstrated that silencing GPX4 induced the expression of HO-1, an effect that was further intensified under RSL3-induced stress. We interpret the significant upregulation of HO-1 in the BAPN group as a compensatory and protective response to high-stress conditions and unregulated heme-mediated oxidative stress. This finding aligns with studies reporting HO-1 as a biomarker of vascular strain in aneurysm models, where its inhibition has been shown to mitigate TAAD-related ECM degradation [35,37,65,66]. However, HO-1 is a double-edged sword; while its induction is intended to be cytoprotective, its excessive activation in an iron-rich environment can have paradoxically harmful effects. By degrading heme, HO-1 releases free ferrous iron (Fe^2+^), which fuels the Fenton reaction and accelerates the production of harmful lipid reactive oxygen species (ROS) [67]. By transcriptionally upregulating SLC7A11 and GPX4, β-OHB strengthens the antioxidant system. This enhancement alleviates the underlying oxidative stress and reduces the pathological need for HO-1 induction, effectively closing the pro-ferroptotic loop that drives tissue degeneration.

The protective effects of β-OHB appear to be mediated through the Nrf2 (*NFE2L2*) signaling pathway. Our silencing experiments confirmed that in the absence of Nrf2, β-OHB loses its capacity to restore GPX4 levels or protect HASMCs from RSL3-induced death. We acknowledge that KE supplementation likely confers additional benefits beyond this pathway. However, our siRNA knockdown of *NFE2L2* and *GPX4* in HASMCs demonstrated that silencing this specific pathway significantly reduces the anti-ferroptotic and cytoprotective effects of β-OHB, even in the context of its broader pleiotropic properties, supporting the β-OHB–Nrf2–GPX4 axis as a crucial mechanism for VSMC preservation and TAAD mitigation. As a potent signaling molecule, β-OHB may regulate Nrf2 in several ways. As an endogenous inhibitor of HDACs [68], β-OHB may increase histone acetylation at the *NFE2L2* promoter, enhancing its transcriptional accessibility and expression. In addition to epigenetic remodeling, β-OHB has been shown to promote the nuclear translocation of Nrf2 in the context of atherosclerosis [69]. Furthermore, β-OHB may also act as a direct signaling ligand for the G protein-coupled receptor HCAR2 (GPR109A), initiating downstream cascades that help regulate redox homeostasis, as suggested by some studies [70,71].

From a translational perspective, the reduction in TAAD incidence from approximately 69% to about 43%, along with an increase in survival rates from roughly 52% to 73%, following KE supplementation, is highly significant. Current pharmacological options for TAAD are primarily limited to anti-hypertensive medications, which may help alleviate mechanical wall stress but do not address the underlying cellular pathology that contributes to medial degeneration. In contrast, nutritional ketosis, achieved through KE, presents a potentially safer and metabolically diverse intervention that targets ferroptosis and may prevent TAAD. Given that ketone esters are already being used in clinical settings for metabolic and cardiovascular conditions, such as heart failure [39], further prospective studies are warranted to assess this strategy as a viable preventative measure for vascular issues like TAAD.

Several limitations of this study deserve acknowledgment. First, while the BAPN model effectively replicates the key histological features of TAAD, it may not fully capture the hemodynamic and genetic complexities associated with human disease, especially in syndromic aortopathies like Marfan or Loeys–Dietz syndromes. Second, although we demonstrated a clear dependence on Nrf2 for β-OHB signaling, the exact upstream molecular events, whether involving epigenetic remodeling or G-protein– coupled receptors, remain to be fully elucidated. Finally, our in vitro mechanistic studies utilized RSL3-mediated GPX4 inhibition as a primary trigger for ferroptosis. This approach may not completely reflect the chronic and intricate events that characterize ferroptosis in the native aortic microenvironment. Future research incorporating conditional, cell-type-specific knockout models will be crucial for validating the translational potential of ketone-based therapies.

## 5. Conclusions

In conclusion, this study demonstrates that ketone ester supplementation can prevent TAAD by inhibiting ferroptosis in the aortic media. The mechanism involves the activation of the Nrf2-GPX4 pathway and a reduction in the negative interaction between GPX4 and HO-1. As a result, β-OHB inhibits iron-mediated lipid peroxidation, protects the aortic media from damage, and preserves the structural integrity of the elastic lamellae. These results suggest that exogenous ketones could be a promising therapeutic strategy for patients who are at high risk of aortic dissection.

## Data Availability

The original contributions presented in this study are included in the article/Supplementary Materials. Further inquiries can be directed to the corresponding author.

## Supplementary Materials

The following supporting information can be downloaded at: https://www.mdpi.com/article/10.3390/cells15090829/s1, Figure S1: Validation of ferroptosis signatures in human ATAA, and dose-dependent effects of β-OHB; Table S1: List of primary antibodies used for Western blotting and IHC; Table S2: List of primers for RT-qPCR.

## Abbreviations

The following abbreviations are used in this manuscript:

β-OHB	β-hydroxybutyrate
4-HNE	4-Hydroxynonenal
ATAA	Acute Thoracic Aortic Aneurysm
BAPN	β-aminopropionitrile
ECM	Extracellular Matrix
FTH1	Ferritin Heavy Chain 1
GPX4	Glutathione Peroxidase 4
GSEA	Gene Set Enrichment Analysis
HASMC	Human Aortic Smooth Muscle Cell
H&E	Hematoxylin and Eosin
HDACs	Histone Deacetylases
*HMOX1*/HO-1	Heme Oxygenase 1
KE	Ketone Ester
MDA	Malondialdehyde
*NFE2L2*/Nrf2	Nuclear Factor Erythroid 2-Related Factor 2
ROS	Reactive Oxygen Species
RSL3	Ras-Selective Lethal 3
scRNA-seq	Single-cell RNA sequencing
SLC7A11/xCT	Solute Carrier Family 7 Member 11
STAAD	Stanford Type A Aortic Dissection
TAAD	Thoracic Aortic Aneurysm and Dissection
TBARS	Thiobarbituric Acid Reactive Substances
TFRC	Transferrin Receptor
VSMC	Vascular Smooth Muscle Cell
VVG	Verhoeff–Van Gieson
