# Supplementary material for: Ketone Ester Attenuates Thoracic Aortic Aneurysm and Dissection by Suppressing Ferroptosis
Source: Cells. 2026 May 1;15(9):829. doi: 10.3390/cells15090829 (PMC13162992; doi:10.3390/cells15090829)
Supplement: Supplementary file 1 [file cells-15-00829-s001.zip › cells-4254958-supplementary/Supplementary materials.pdf]

Table S1. List of antibodies used for Western blotting and IHC

Antibody	Manufacturer	Catalog no.
GPX4	Abcam	Ab125066
HO-1	Proteintech	10701-1-AP
SLC7A11	CST	12691S
FTH1	Proteintech	11682-1-AP
4-HNE	Invitrogen	MA5-27570
β-actin	Santa Cruz	Sc-47778
β-tubulin	Proteintech	66240-1-Ig
GAPDH	Santa Cruz	Sc-32233

Table S2. List of primers for RT-qPCR

Gene		**Primer sequence (Forward)**	**Primer sequence (Reverse)**
GPX4	Human	GAGGCAAGACCGAAGTAAACTAC	CCGAACTGGTTACACGGGAA
HMOX1	Human	AAGACTGCGTTCCTGCTCAAC	AAAGCCCTACAGCAACTGTCG
SLC7A11	Human	TCTCCAAAGGAGGTTACCTGC	AGACTCCCCTCAGTAAAGTGAC
ACTB	Human	AAACTGGAACGGTGAAGGTGA	CTCGGCCACATTGTGAACTT
GAPDH	Human	GAAAGCCTGCCGGTGACTAA	GCATCACCCGGAGGAGAAAT
Gpx4	Mouse	GATGGAGCCCATTCCTGAACC	CCCTGTACTTATCCAGGCAGA
Hmox1	Mouse	AAGCCGAGAATGCTGAGTTCA	GCCGTGTAGATATGGTACAAGGA
Slc7a11	Mouse	GGCACCGTCATCGGATCAG	CTCCACAGGCAGACCAGAAAA
Actb	Mouse	GGCTGTATTCCCCTCCATCG	CCAGTTGGTAACAATGCCATGT
Gapdh	Mouse	AGGTCGGTGTGAACGGATTTG	TGTAGACCATGTAGTTGAGGTCA

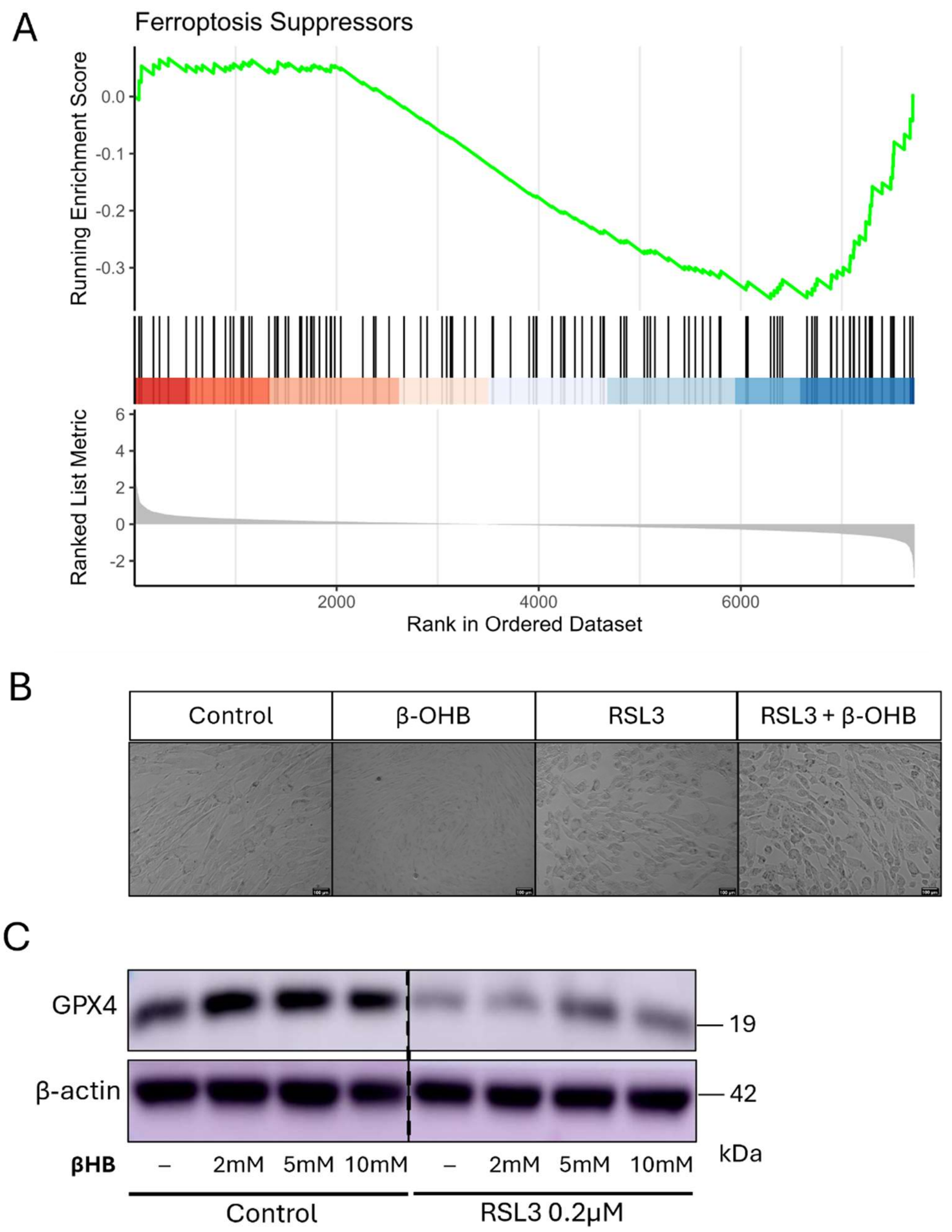

Supplementary Figure S1.Validation of ferroptosis signatures in human ATAA, and dose-dependent effects of β-OHB. (**A**) Gene Set Enrichment Analysis (GSEA) plot for "Ferroptosis Suppressors" in vascular smooth muscle cell (SMC) clusters derived from single-cell RNA sequencing (scRNA-seq) of human control and Acute Thoracic Aortic Aneurysm (ATAA) samples (GSE155468). (**B**) Representative bright-field microscopy images of Human Aortic Smooth Muscle Cells (HASMCs) illustrating morphological changes across Control, β-OHB, RSL3, and RSL3 + β-OHB treatment groups. Scale bar = 100 µm. Treatments: β-OHB (5 mM), RSL3 (50 nM) for 24 hours. (**C**) Representative Western blot analysis of GPX4 protein expression in HASMCs demonstrating the dose-dependent effects of β-OHB (2 mM, 5 mM, 10 mM, 24 hours) under baseline conditions and following acute RSL3 challenge (0.2 µM, 2 hours).
